# Jamb and Jamc Muscle in on Myoblast Fusion

**DOI:** 10.1371/journal.pbio.1001217

**Published:** 2011-12-13

**Authors:** Caitlin Sedwick

**Affiliations:** Freelance Science Writer, San Diego, California, United States of America

## Abstract

Jamb and Jamc are an essential cell surface receptor pair that interact to drive fusion between muscle precursor cells during zebrafish development.

**Figure 1 pbio-1001217-g001:**
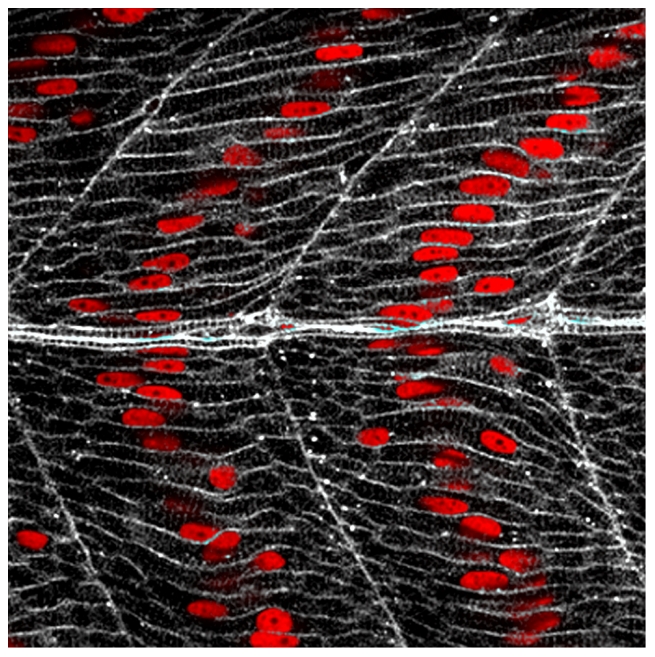
Loss of the receptor protein Jamb in zebrafish blocks muscle precursor fusion, resulting in mononuclear fast-twitch skeletal muscle fibers. The single nucleus within each mutant fiber (in red) becomes centrally located, creating a striking pattern that repeats along the trunk and tail.

[Fig pbio-1001217-g001]Dashing down a track, sprinters rely on the special properties of the fast-twitch muscle fibers interspersed throughout their skeletal muscles. Meanwhile, long-distance runners are more concerned with performance of the slow-twitch muscle fibers in those same muscles. In humans, these fibers differ in the number of mitochondria they contain, but both types of muscle are formed from a cellular syncytium: many muscle cells fuse together into a multinucleate mass. It's known that these syncytia form during embryonic development, though how they do so and which proteins mediate the fusion of individual vertebrate muscle cells has remained a mystery. In this month's *PLoS Biology*, Gareth Powell and Gavin Wright shed light on these questions by identifying a pair of cell surface receptors essential for the development of fast-twitch muscle syncytia.

One of the first hurdles for the formation of a syncytium is the identification and mutual recognition of prospective fusion partners. In the fruit fly *Drosophila melanogaster*, this is accomplished via the expression of a special pair of cell surface receptors on the fusing muscle cells, which are required for fusion to occur. But in vertebrates, no such pair has been identified. Instead, several surface proteins appear to contribute to the recognition process, and although it has been proposed that they—and potentially other surface proteins—may act cooperatively to drive recognition and subsequent fusion, this has not been definitively shown.

To identify additional proteins that might participate in the recognition and fusion process, Powell and Wright chose to work in zebrafish, an organism that's easily genetically manipulated and whose embryonic development can be observed from start to finish because it takes place in transparent eggs. Like humans, zebrafish have both fast- and slow-twitch muscle fibers. However, while their fast-twitch fibers fuse into syncytia, their slow-twitch fibers do not. The authors therefore screened a library of zebrafish surface proteins using an assay that is optimized for detecting the brief and often weak interactions that take place between extracellular surface proteins. Their screen identified a pair of receptors, Jamb and Jamc, which are expressed at high levels on developing muscle cells, or myoblasts, during the time when syncytium creation is taking place in zebrafish embryos. Significantly, these proteins are found on fast-twitch but not slow-twitch myoblasts.

To explore whether Jamb and Jamc are required for myoblast fusion, the authors obtained zebrafish embryos lacking functional versions of each individual protein. Mutant animals lacking either protein failed to form syncytia in their fast-twitch muscle fibers; instead, individual myoblasts remained distinct and formed separate, mononucleate muscle fibers as the muscle developed. This finding suggests that Jamb and Jamc drive the recognition of neighboring cells and mediate cell fusion.

How do the proteins accomplish this? It's possible that the two proteins must bind each other in order to achieve recognition, or alternatively, that recognition could be driven by Jamb–Jamb or Jamc–Jamc binding interactions. While Powell and Wright showed that all three interactions are possible in vitro, the Jamb–Jamc interaction is by far the strongest of the three, suggesting it's this one that drives fusion. Indeed, the authors found that fast-twitch myoblasts could only fuse when one cell expresses Jamb and the other Jamc.

The authors' findings show that Jamb and Jamc are required for fusion, but do not show whether this pair of receptors is sufficient for the process to proceed. In fact, when the authors expressed Jamb and Jamc together on cells other than fast-twitch myoblasts, those cells did not fuse, suggesting the involvement of additional, unidentified factors. This result is consistent with the fact that expression of these proteins is not limited to myoblasts: Jamc is found at low expression levels throughout the developing embryo, and in mammals, both proteins are found in tissues such as the vascular endothelium. Endothelial cells do not undergo fusion; instead, other researchers have shown that Jamb and Jamc act to promote adhesion of neighboring endothelial cells. Therefore, although Jamb and Jamc are required for fast-twitch myoblast fusion, they are not by themselves sufficient to promote it.

Powell and Wright speculate that fusion depends on the appropriate cellular context, which is probably determined by the presence of other proteins in fusing cells. Exactly what that cellular context may be is something the authors plan to address in future work. Ultimately, because fusion is not limited to muscle tissues, the study of this receptor pair could yield important insights that go beyond muscle development.

**Powell GT, Wright GJ (2011) Jamb and Jamc Are Essential for Vertebrate Myocyte Fusion. doi:10.1371/journal.pbio.1001216**

